# Antibody-drug conjugates for primary brain tumors

**DOI:** 10.3389/fonc.2026.1811494

**Published:** 2026-05-08

**Authors:** L. Nicolas Gonzalez Castro, Sandro Santagata

**Affiliations:** 1 Center for Neuro-Oncology, Dana-Farber Cancer Institute, Boston, MA, United States; 2 Department of Neurology, Brigham and Women’s Hospital, Boston, MA, United States; 3 Laboratory of Systems Pharmacology, Harvard Medical School, Boston, MA, United States; 4 Department of Pathology, Brigham and Women’s Hospital, Boston, MA, United States

**Keywords:** antibody-drug conjugates, atypical teratoid/rhabdoid tumors, craniopharyngioma, ependymoma, glioblastoma, primary brain tumors

## Abstract

Antibody-drug conjugates (ADCs) enable targeted delivery of cytotoxic or immunomodulatory payloads to cancer cells while relatively sparing healthy tissues. Interest in using ADCs for the treatment of primary brain tumors is growing, as these are frequently infiltrative with tumor cells coexisting with healthy glial and neural tissue. Emerging clinical and translational data from studies of ADCs in primary brain tumors and brain metastases provide evidence of blood-brain barrier penetration and intracranial anti-tumor activity. Proteomic and transcriptomic profiling methods, including RNA sequencing and immunohistochemistry have uncovered clinically actionable ADC targets in primary brain tumors that are not detected by DNA-based next-generation sequencing alone. These targets (HER2, TROP2, FOLR1, CLDN6, HER3, B7-H3) are expressed across both common tumor types (gliomas, meningioma) and rare primary brain tumors (ependymoma, craniopharyngioma, atypical teratoid/rhabdoid tumors). Leveraging label-extension strategies, ADCs already approved for systemic cancers may be rapidly evaluated and translated into neuro-oncology practice. Here we summarize the current evidence supporting ADC use in primary brain tumors and highlight key challenges and future directions for the further development of this therapeutic approach in neuro-oncology.

## Introduction

Primary central nervous system (CNS) tumors are the leading cause of cancer in children up to 14-years old, the second most common in adolescents and young adults up to 39, and the seventh most common cause in those 40 or older (1). In addition to constituting a major source of mortality (ranking 4^th^, 5^th^ and 9^th^ in the above age groups, respectively), CNS tumors are a major cause of disability due to tumor location and the neurological sequalae of treatment (1). Despite advances in surgery, radiation, and chemotherapy over the past 20 years, most malignant primary brain tumors show limited durable response to standard therapies and remain associated with poor outcomes. Infiltrating gliomas – the most common and aggressive primary brain tumors – inevitably recur after treatment (2) and have shown minimal benefit from cancer treatment strategies that have transformed outcomes in many systemic cancers, including immunotherapy (3).

By enabling the targeted delivery of potent therapeutic payloads while generally sparing normal tissues, antibody drug conjugates (ADCs) represent a promising therapy strategy for primary brain tumors which are characterized by malignant cells intermingled with neural and glial structures. In the following sections, we provide an overview of (i) the structure and mechanism of action of antibody drug conjugates (ADCs); (ii) the emerging evidence supporting blood-brain barrier penetration and central nervous system activity of ADCs, largely obtained from clinical studies in patients with CNS metastases; (iii) the available, albeit limited, clinical experience with ADCs in primary brain tumors; and (iv) the identification of clinically actionable, and often overlooked ADC targets expressed in primary brain tumors that may enable new therapeutic and label-extension opportunities in neuro-oncology.

## ADC development and mechanisms of action

The therapeutic window of a chemotherapeutic agent is defined as the dosing range that permits effective treatment without unacceptable toxicity. The concept of a “magic bullet” – a therapy capable of eliminating diseased cells while sparing normal tissues – can be traced to the work of Paul Ehrlich’s in the early 1900s (4, 5). Although cytotoxic and targeted therapies can effectively kill cancer cells, their clinical utility is often limited by off-target effects on healthy tissues. Advances in antibody engineering, including chimeric, humanized and fully human monoclonal antibodies (mAbs) have enabled more precise targeting of cell-surface antigens with reduced immunogenicity (6, 7). In 2000, gemtuzumab ozogamicin, a humanized anti-CD33 antibody linked to the cytotoxic antibiotic calicheamicin, became the first FDA-approved ADC following evaluation in acute myeloid leukemia (8). Since then, 14 ADCs targeting diverse antigens have been approved for the treatment of solid and hematological malignancies (**Table 1**).

**Table 1:** FDA approved ADCs as of 2025.

ADC (brand name)	Target	Antibody	Linker	Payload	Indications	Year of FDA Approval
Gemtuzumab Ozogamicin (Mylotarg)	CD33	Gemtuzumab	Hydrazone	Calicheamicin	AML	2000; 2017
Brentuximab Vedotin (Adcetris)	CD30	Brentuximab	mc-VC-PABC	MMAE	HL, ALCL, PTCL	2011
Trastuzumab Emtansine (Kadcyla)	HER2	Trastuzumab	SMCC	DM1	HER2+ Breast Cancer	2013
Inotuzumab Ozogamicin (Besponsa)	CD22	Inotuzumab	Hydrazone	Calicheamicin	B-cell precursor ALL	2017
Moxetumomab Pasudotox (Lumoxit)	CD22	Moxetumomab	mc-VC-PABC	PE38	HCL	2018*
Polatuzumab Vedotin (Polivy)	CD79b	Polatuzumab	mc-VC-PABC	MMAE	DLBCL	2019
Enfortumab Vedotin (Padcev)	Nectin-4	Enfortumab	mc-VC-PABC	MMAE	Urothelial Cancer	2019
Trastuzumab Deruxtecan (Enhertu)	HER2	Trastuzumab	Tetrapeptide	DXD	HER2+/HER2-low Breast, Gastric and Lung Cancers	2019
Sacituzumab Govitecan (Trodelvy)	TROP2	Sacituzumab	CL2A	SN-38	TNBC, HR+/HER2- Breast Cancer, Urothelial Cancer	2021
Loncastuximab Tesirine (Zynlonta)	CD19	Loncastuximab	mc-VC-PABC	PBD SG3199	DLBCL	2021
Tisotumab Vedotin (Tivdak)	TF	Tisotumab	mc-VC-PABC	MMAE	Cervical Cancer	2021
Mirvetuximab Soravtansine (Elahere)	FRα	Mirvetuximab	Sulfo-SPDB	DM4	Ovarian Cancer	2022
Datopotamab Deruxtecan (Datroway)	TROP-2	Datopotamab	Tetrapeptide	DXD	HR+/HER2- Breast Cancer; EGFR-mutated NSCLC	2025
Telisotuzumab Vedotin (Emrelis)	c-Met	Telisotuzumab	mc-VC-PABC	MMAE	c-Met+ NSCLC	2025

**Figure 1:**
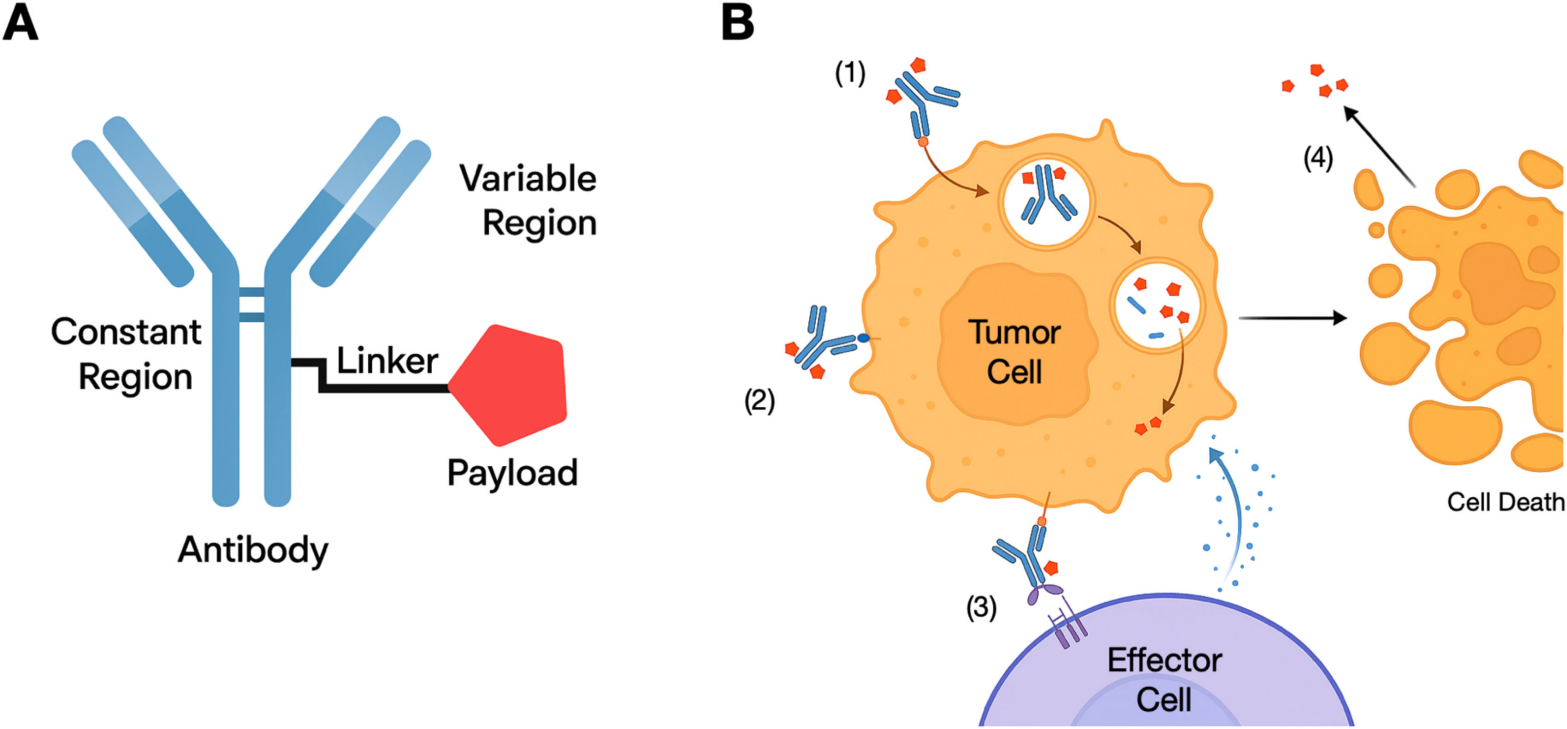
Antibody-drug conjugate structure and mechanisms of action. **(A)** The structure of an antibody-drug conjugates includes an antibody targeting a cancer cell surface molecule, a therapeutic payload and a linker molecule. **(B)** Antibody-drug conjugates can act on cancer cells (1) via targeted delivery of the payload, causing cytotoxicity; (2) via blockade of a pro-survival receptor (e.g., HER2); (3) via induction of antibody-dependent cellular cytotoxicity; or (4) via release of cytotoxic payload after cell death impacting nearby tumor cells (bystander effect).

Despite the molecular diversity across approved agents, ADCs share a common structure composed of 3 elements: (1) a targeting antibody directed against a tumor-associated antigen, (2) a linker connecting the antibody to the payload, (3) and a potent cytotoxic or immunomodulatory payload (**Figure 1A**). Antibody-mediated targeting allows the selective delivery of highly potent payloads to cancer cells expressing a surface antigen of interest, thereby enabling the use of agents with narrow therapeutic windows that would otherwise demonstrate intolerable toxicity if administered systemically. The drug-antibody ratio (DAR) specifies the number of therapeutic molecules that are coupled to a single antibody. The DAR, ranges between 1 and 8, is optimized based on potency, stability and pharmacokinetic considerations (9). These design features are particularly relevant for brain tumors, where infiltrative growth and limited tolerance for off-target toxicity limit the use of conventional therapeutics.

Multiple considerations come into play for the design of linker molecules in ADCs. Linkers must remain stable in systemic circulation while efficiently releasing payload inside target cells, often in response to specific intracellular conditions such as acidic endosomes pH or protease activity. Non-cleavable linkers, which release the therapeutic payload only after antibody degradation in lysosomes can minimize toxicity to non-target cells (10). Linker hydrophilicity influences solubility and pharmacokinetics, while chemical stability preserves payload activity during conjugation and storage (9). Mechanistically, ADCs induce cancer cell death via targeted payload delivery but may also exert effects through pro-survival receptor blockade, antibody-dependent cellular cytotoxicity (ADCC), or bystander killing of neighboring tumor cells following payload release from dying cells (9, 11, 12) (**Figure 1B**).

Based on their size and polarity, ADCs would be predicted to have limited penetration across the blood brain barrier (BBB) and minimal activity in CNS tumors (13). However, emerging data challenge this assumption, demonstrating meaningful activity of several ADCs in patients with brain metastases and proving a rationale for evaluating these agents in primary brain tumors, as discussed in the following sections.

## CNS activity of ADCs

ADCs are large macromolecules (~150 kDa), and passive diffusion across an intact blood-brain barrier (BBB) typically favors small (up to approximately 500 Da) lipophilic molecules. As such a large biologic such as an ADC is not expected to cross efficiently under normal conditions (14, 15). In addition, the hydrophilic and polar properties of ADCs further limit transcellular BBB permeability (11). However, BBB permeability is heterogeneous in the context of CNS tumors, and tumor-associated vascular abnormalities and neurovascular functional disruption may permit intracranial exposure. For instance, glioblastomas grow tortuous blood vessels with abnormal endothelial-pericyte interactions leading to a highly permeable blood-tumor barrier (16).

Despite the theoretical BBB penetration constraints noted above, accumulating clinical evidence demonstrates that several ADCs achieve meaningful intracranial activity in patients with brain metastases (**Table 2**), challenging the assumption that the BBB precludes therapeutic efficacy. Contributing mechanisms likely include localized BBB disruption within enhancing tumor regions, receptor-mediated uptake and intracellular trafficking, and diffusion of membrane-permeable payloads capable of bystander effects within the tumor microenvironment. Emerging delivery strategies (focused ultrasound–mediated BBB disruption) also support the feasibility of augmenting intracranial exposure of large biologics. Thus, these observations provide strong indications that ADCs may exert therapeutic activity within the CNS and support their evaluation in gliomas and other CNS tumors expressing relevant ADC targets.

**Table 2:** Select studies providing evidence of ADC activity against brain metastases.

ADC	Target	Study	Population	ORR for Brain Metastases	Reference
Trastuzumab Emtansine (T-DM1)	HER2	KAMILLA (Phase IIIb; exploratory *post hoc* analysis)	HER2+ breast cancer; 398 patients with brain metastases (126 with measurable disease)	21.4%	(17)
Trastuzumab Deruxtecan (T-DXd)	HER2	DESTINY-Breast01 (Phase II; exploratory *post hoc* analysis)	HER2+ breast cancer; 24 patients with brain metastases (14 with measurable disease)	50.0%	(18)
Trastuzumab Deruxtecan (T-DXd)	HER2	DESTINY-Breast03 (Phase III; exploratory *post hoc* analysis)	HER2+ breast cancer; 82 patients with brain metastases	63.9%	(19)
Trastuzumab Deruxtecan (T-DXd)	HER2	TUXEDO-1 (Single arm phase II)	HER2+ breast cancer with brain metastases	73.3%	(20)
Trastuzumab Deruxtecan (T-DXd)	HER2	DEBBRAH (Single-arm phase II trial)	HER2+ breast cancer with brain metastases, including leptomeningeal metastases	46.2%	(21)
Patritumab Deruxtecan (HER3-DXd)	HER3	TUXEDO-3 (phase II)	Breast cancer brain metastases, NSCLC brain metastases, and leptomeningeal metastases from advanced solid tumors	11.0%	(23)
Patritumab Deruxtecan (HER3-DXd)	HER3	HERTHENA-Lung01 (phase II)	Advanced EGFR-mutated NSCLC	33.3%	(24)
Ifanatamab Deruxtecan (I-DXd)	B7-H3	IDeate-Lung01 (phase II)	Extensive stage SCLC	46.2%	(25)

AML: acute myeloid leukemia; HL: Hodgkin lymphoma; ALCL: anaplastic large cell lymphoma; PTCL: peripheral T cell lymphoma; HER2: human epidermal growth factor receptor 2; ALL: acute lymphoblastic leukemia; HCL: hairy cell leukemia; DLBCL: diffuse large B-cell lymphoma; TNBC: triple negative breast cancer; HR: hormone receptor; EGFR: epidermal growth factor receptor; NSCLC: non-small cell lung cancer; *Moxetumomab pasudotox was discontinued in 2023.

In patients with HER2-positive breast cancer and brain metastases, trastuzumab emtansine (T-DM1) demonstrated an intracranial overall response rate (ORR) of 21.4% among 126 patients with measurable metastases in the single-arm phase IIIb KAMILLA trial (17). Trastuzumab deruxtecan (T-DXd) demonstrated substantially higher intracranial activity, with ORRs of 50% and 63.9% in exploratory *post hoc* analyses of the phase II DESTINY-Breast01 (18) and phase III DESTINY-Breast03 (19) trials, respectively, and ORRs of 73.3% and 46.2% in the single-arm phase II TUXEDO-1 (20) and DEBBRAH (21) studies. In HER2-mutant non-small-cell lung cancer (NSCLC), T-DXd demonstrated an ORR of 54.5% among 33 patients with brain metastases in the phase II DESTINY-Lung01 trial (22).

More recently, patritumab deruxtecan (HER3-DXd), an ADC targeting HER3/Erb3, has shown intracranial activity across multiple tumor types. In the phase II TUXEDO-3 trial, HER3-DXd demonstrated an overall intracranial ORR of 11% in patients with breast cancer brain metastases, NSCLC brain metastases, and leptomeningeal disease (23), and an ORR of 33.3% in patients with EGFR-mutant NSCLC in the HERTHENA-Lung01 phase II study (24). Similarly, ifanatamab deruxtecan (I-DXd), an ADC against B7-H3, demonstrated an intracranial response rate of 46.2% in patients with extensive-stage small cell lung cancer (SCLC) enrolled in the IDeate-Lung01 phase II trial (25).

Although the precise mechanisms of ADC response remain incompletely defined, contributing factors likely include BBB disruption, receptor-mediated uptake, and diffusion of payloads capable of bystander effects within the tumor microenvironment. Together, these clinical responses provide strong proof-of-principle that ADCs can have therapeutic activity within the CNS and support their evaluation in gliomas and other primary brain tumors expressing relevant ADC targets, as reviewed in the subsequent sections.

## Response to ADCs in gliomas

Infiltrating gliomas – including glioblastoma (GBM), isocitrate-dehydrogenase (IDH) mutant glioma, and H3 K27-altered diffuse midline glioma (DMG) – remain highly resistant to standard therapies. In GBM, alterations in EGFR – including gene amplification and the mutant splice variant EGFRvIII – are present in approximately 50% of tumors and represent rational therapeutic targets (see **Table 3**) (26, 27). EGFRvIII is present in approximately 25% of glioblastomas and is largely tumor-specific, making it an attractive candidate for ADC-based targeting (28). Depatuxizumab mafodotin (Depatux-M) is an ADC combining the anti-EGFRvIII monoclonal antibody depatuxizumab and the microtubule inhibitor monomethyl auristatin F (MMAF) via a non-cleavable linker (27, 29). The M12–356 phase I trial evaluated Depatux-M in patients with newly diagnosed and progressive GBM also receiving radiation and temozolomide, as well as in progressive GBM patients receiving Depatux-M as monotherapy (29). Among patients receiving Depatux-M as monotherapy, the ORR was 6.8% (29). The most common adverse event (occurring in 87-92% of patients) was ocular toxicity (corneal epitheliopathy), attributed to an on-target, off-tumor effect resulting from binding to wildtype EGFR, which is constitutively expressed in cornea epithelium (29, 30).

**Table 3:** Select studies evaluating ADCs for the treatment of glioblastoma.

ADC	Target	Study	Population	Outcomes	Reference
Depatuxizumab Mafodotin (Depatux-M)	EGFRvIII/EGFR	M12-356 (Phase I)	Progressive *EGFR*-amplified GBM receiving Depatux-M monotherapy (Cohort C expansion)	ORR 6.8%; ocular toxicity in > 80% of patients	(29)
Depatuxizumab Mafodotin (Depatux-M)	EGFRvIII/EGFR	INTELLANCE-1 (Phase III)	Newly diagnosed *EGFR*-amplified GBM receiving chemoradiotherapy +/- Depatux-M	PFS 8.0 vs 6.3 months in patients receiving Depatux-M (HR 0.84)	(31)
Depatuxizumab Mafodotin (Depatux-M)	EGFRvIII/EGFR	INTELLANCE-2 (Phase II)	*EGFR*-amplified GBM at first progression receiving Depatux-M +/- TMZ	OS 9.6 vs 8.2 months in patients receiving Depatux-M + TMZ (HR 0.66)	(32)
AMG-595	EGFRvIII	AMG-595 (Phase I)	Patients with progressive glioblastoma and EGFRvIII expression	ORR 6%	(33)

In the pivotal phase III INTELLANCE-1 trial (31), the addition of Depatux-M to standard radiation and chemotherapy did not improve overall survival (OS) in patients with newly diagnosed glioblastoma, despite a modest improvement in progression-free survival (8.0 vs 6.3 months, HR 0.84). In the phase II INTELLANCE-2 trial, Depatux-M was evaluated at first recurrence, either alone or in combination with temozolomide (TMZ) (32). The combination arm achieved a modest OS benefit (9.6 vs 8.2 months; HR 0.66, p = 0.01), with a trend toward great benefit in tumors without methylation of the O6-methylguanine-DNA methyltransferase (MGMT) promoter, which are expected to draw less benefit from TMZ (32). Given the significant ocular toxicity and the lack of durable clinical benefit, Depatux-M has not undergone further clinical development.

Other EGFR-targeting ADCs – including losatuxizumab (anti-EGFR mAb) vedotin, serclutamab (anti-EGFR mAb) talirine and AMG-595 (anti-EGFRvIII mAb conjugated with emtansine) – have been evaluated in early-phase clinical trials. While these agents demonstrated improved tolerability relative to Depatux-M, none showed a clear signal of efficacy in GBM (33–35). Together, these studies highlight key biological and technical challenges to ADC developed for targeting GBM, including target heterogeneity (36, 37), antigen loss, and payload-associated toxicity. In regard to target heterogeneity, single-cell RNA-sequencing studies have demonstrated that the intra-tumoral heterogeneity of GBM is characterized by four dominant cellular states with gene expression programs that demonstrate plasticity and show preferential localization within the tumor architecture (36, 38). Effective targeting of GBM with ADCs, would require the use of ADCs targeting multiple cellular states or at least targeting those cell states that demonstrate the highest degree of plasticity, something that could be achieved by the use of payloads that could degrade transcription factors that drive specific cell states, such as proteolysis-targeting chimeras (PROTACs) (39).

Beyond GBM, ADCs have undergone preclinical evaluation in H3 K27-altered DMG. These tumors display high expression of B7-H3 (40), and the surface ganglioside GD2 has also emerged as an additional candidate target. GD2-targeting ADCs have shown *in vitro* cytotoxicity and prolonged survival in orthotopic DMG models (41), supporting further investigation of ADC strategies in this treatment refractory malignancy.

As is common with targeted therapies, resistance to ADCs arises through multiple mechanisms, including intratumoral heterogeneity and loss or downregulation of targets antigen, alterations in antibody internalization or intracellular trafficking, and lysosomal dysfunction. Tumor cells can also develop resistance to specific classes of payloads through drug efflux pumps, mutations in drug targets, or development of adaptive states, which may be particularly relevant in gliomas which are characterized by highly plastic lineage states. Collectively, these resistance mechanisms highlight the need for integrative spatial diagnostics that evaluate target expression and engagement of resistance mechanisms (42, 43).

## Identifying primary brain tumors responsive to ADC therapy

Although molecular alterations increasingly inform primary brain tumors classification, routine characterization methods, including DNA next-generation sequencing, do not reliably identify therapeutically actionable targets, and many recurrent driver alterations remain difficult to drug. In contrast, ADC efficacy depends on target abundance and cell surface localization, rather than underlying genotypes. In this context, a recent systematic analysis integrating information from RNA sequencing, proteomics and immunohistochemistry revealed high expression of multiple ADC targets in ependymoma, meningioma, and craniopharyngioma, with more moderate expression of select targets in adult infiltrating gliomas, including glioblastoma and IDH-mutant glioma (44). The presence of these targets across multiple CNS tumor types highlights opportunities for label extension of FDA-approved ADCs from breast, ovarian, lung, and gastrointestinal cancers into rare primary brain tumors, independent of specific genomic alterations.

Distinct expression patterns inform target prioritization across tumor subtypes. Papillary craniopharyngiomas broadly express TROP2 throughout the tumor compartment, whereas adamantinomatous craniopharyngioma (ACP) exhibit HER2 and TROP2 expression largely confined to characteristic epithelial whorls marked by nuclear accumulation of mutant β-catenin (45). Given these β-catenin-active tumor-organizing regions are largely non-proliferative (46), effective targeting may require payloads capable of acting in this cell state, although bystander effects may still provide therapeutic benefit by eliminating adjacent proliferative cells that depend on paracrine signaling from these β-catenin-active hubs. Atypical teratoid/rhabdoid tumors (AT/RT), as well as extra-cranial malignant rhabdoid tumors, express variable levels of CLDN6 (47), a validated ADC target in a subset of high-grade serous ovarian cancer (48). Ependymomas, spanning histologic subtypes (49) including myxopapillary ependymoma, also demonstration variable expression of HER2. Cell-surface targets may be particularly attractive in ependymoma, as that many tumors lack actionable genomic drivers, and recurrent alterations such as MYCN-amplification, ZFTA-fusions, and YAP1-fusions are not currently targetable (50). Transcriptomic analyses further indicate expression of folate-receptor alpha (FOLR1), the target of an FDA-approved ADC, in ependymoma and meningioma.

Publicly available data resources such as CPTAC (51) have been instrumental in accelerating target discovery and validation across both rare and common CNS tumor types by enabling integrated analysis of proteomic, genomic and clinical data. Emerging spatial proteomics and spatial transcriptomics technologies extend this framework by revealing how target expression varies across distinct tumor niches. Broad access to these data through platforms such as cBioPortal (52) will further empower the research community to identify actionable cell-surface targets, validate antibodies, and inform clinical testing strategies.

As the number of FDA-approved ADCs continues to expand and clinical activity is explored in primary brain tumors, increasingly precise deployment of these agents will be required. Clinical Laboratory Improvement Amendments (CLIA)-ready multiplexed imaging assays offer a tissue-sparing approach to quantitatively evaluate multiple ADC targets within a single biopsy (53, 54). A single FFPE section could be assayed for HER2, TROP2, FOLR1, CLDN6, HER3, B7-H3 plus a minimal context panel (e.g., SOX2, OLIG2, GFAP, CD45, CD68) to interpret tumor versus immune compartments and spatial heterogeneity. Such assays can support ADC selection based by integrating information on target expression level, subcellular localization, and regional heterogeneity, as well as tumor-intrinsic and tumor-immune microenvironment features associated with response.

Although some intracranial tumors arise outside an intact BBB and intracranial responses have been observed in metastatic disease, additional delivery strategies, including intrathecal or intra-cerebrospinal fluid administration and focused ultrasound-mediated BBB disruption will warrant further investigation. Moreover, despite indirect evidence that therapeutically relevant intracranial exposure is achievable, optimization of payload properties, linker chemistry, and antibody design for CNS delivery may be required to maximize ADC efficacy in primary brain tumors. A relevant issue is addressing the binding site barrier phenomenon, where high-affinity antibodies tend to tightly bind tumor cells adjacent to blood vessels, failing to penetrate into the tumor core. The challenge of the binding site barrier could potentially be overcome by co-administration of the ADC and elements of its targeting antibody to allow for transient competitive inhibition that can allow for deeper penetration of the ADC (55).

## Discussion

Advances in ADC design are ushering a new era for the treatment of systemic malignancies (56), with growing evidence of responses in patients with brain metastases. These observations have motivated the emergent evaluation of ADCs for the treatment of primary brain tumors. The present article aims to provide a structured discussion of how this novel therapeutic approach is beginning to make inroads for the treatment of glioblastoma and other primary brain tumors – starting from the recognition of intracranial activity in brain metastases, to initial observations on primary brain tumor responses based on available clinical trials. In addition, we aim to highlight novel approaches for the identification of ADC targets in primary brain tumors. Although clinical trials of EGFR and EGFRvIII-targeting ADCs in glioblastoma have demonstrated only limited efficacy, they nonetheless provide proof of principle that ADC-based strategies can be deployed against infiltrating CNS tumors. While cellular heterogeneity remains a fundamental barrier to durable response in gliomas (37) and other solid tumors (57), recent systematic profiling approaches have identified additional, clinically actionable ADC targets across a range of primary brain tumors (44), supporting feasibility of leveraging existing ADCs for CNS indications.

As ADC development for primary brain tumors progresses, several barriers must be addressed, including (1) limited BBB penetration, (2) target heterogeneity and antigen downregulation (particularly in glioblastoma, where EGFRvIII expression is often lost at recurrence (58)), and (3) systemic toxicity resulting from low level antigen expression in normal tissues. Potential avenues to address these include (1) more accurate pre-clinical models of BBB biology (59), improved delivery methods (e.g., convection-enhanced delivery, BBB-disruption via focused ultrasound (60), strategies that leverage receptor-mediated transcytosis for brain delivery of therapeutics (61), or intratumoral/intrathecal delivery), (2) ADC combinations targeting multiple antigens, targeting cancer stem cells (62) or the immunosuppressive components of the tumor microenvironment (63), and longitudinal monitoring of target availability via serial tissue or liquid biopsies, and (3) continued optimization of ADC design to enhance target specificity and payload delivery. The induction of immunogenic cell death by ADCs and its resulting anti-tumor immune activation (64), constitutes another approach to overcome resistance mechanisms based on intratumoral heterogeneity, a mechanism that has also been observed with other therapeutic approaches that modulate the tumor microenvironment (65).

In regard to safety, besides the ocular toxicity observed with anti-EGFRvIII ADCs (30), other frequent systemic toxicities due to on-target/off-tumor effects, including hematologic, gastrointestinal, dermatologic, respiratory and peripheral nerve toxicities, have been described with approved ADCs (66) and remain an important consideration in clinical development. In addition, a recent analysis suggests that many instances of ADC toxicity are independent of target antigen expression and seem to be associated with payload deployment in healthy tissues (67), highlighting the challenge of effective ADC design. Payload-binding selectivity enhancers (PBSEs) provide an approach to minimize this toxicity and increase the therapeutic index of ADCs (68, 69).

Progress in this field will also require the development of predictive biomarkers that capture target expression, immune contexture, and biophysical features of the tumor microenvironment that influence payload release and bioavailability. Implementation of CLIA-ready multiplexed imaging assays like the ones we describe here could enable quantitative, tissue sparing assessment of multiple ADC targets within a single biopsy, facilitating patient stratification in ways that exceed conventional single marker immunohistochemistry and semi-quantitative visual scoring. For example, concurrent profiling of HER2, TROP2, FOLR1 or other ADC targets in a primary brain tumor specimen could streamline therapeutic decision-making and accelerate deployment of targeted treatment options. Realizing this next generation of companion diagnostics will require continued innovation in pathology laboratories and workflows.

## Conclusion

In a general sense, ADC development reflects a shift away from single-agent paradigms toward modular, mechanism driven therapeutic platforms, in which antibodies can be paired with distinct payloads and linkers chemistries to optimize efficacy, CNS delivery, and safety. Viewed as an engineering framework rather than a fixed therapeutic class, ADCs offer opportunities for iterative refinement and rational combination strategies, including co-targeting of multiple antigens, modulation of antigen presentation, integration with immune-directed therapies, and exploration of tumor metabolic vulnerabilities.

Importantly, the absence of brain-tumor-specific ADC targets should not be viewed as a limitation. Instead shared expression of well-validated targets such as HER2, FOLR1, TROP2, and CLDN6 across systemics and CNS tumors enables rapid label-extension strategies that bypass the slow pace of *de novo* drug development in neuro-oncology. Timely reporting of both positive and negative results from such efforts, particularly in rare brain tumors, will accelerate progress toward effective treatment strategies and may yield insights applicable to more common malignancies within and beyond the CNS.

Collectively, these advances will bring the field closer to fulfilling the century-old vision of a “magic bullet” for brain cancer, one that overcomes genetic heterogeneity by exploiting surface-accessible tumor vulnerabilities to guide precision therapy.
